# Prognostic significance of peripheral CD8+CD28+ and CD8+CD28− T cells in advanced non-small cell lung cancer patients treated with chemo(radio)therapy

**DOI:** 10.1186/s12967-019-2097-7

**Published:** 2019-10-17

**Authors:** Chao Liu, Wang Jing, Ning An, Aijie Li, Weiwei Yan, Hui Zhu, Jinming Yu

**Affiliations:** 1 Department of Oncology, Renmin Hospital of Wuhan University, Wuhan, 430060 China; 2 Department of Radiation Oncology, Shandong Cancer Hospital and Institute, Shandong First Medical University and Shandong Academy of Medical Sciences, Jinan, 250117 Shandong China; 3 Department of Radiation Oncology, Affiliated Hospital of Academy of Military Medical Sciences, Beijing, 100071 China; 4 Weifang Medical University, Weifang, 261053 Shandong China

**Keywords:** CD28, Prognostic value, Squamous cell carcinoma, Adenocarcinoma, Peripheral blood

## Abstract

**Background:**

Noninvasive prognostic biomarkers are needed for advanced non-small cell lung cancer (NSCLC) patients with different histological types to identify cases with poor survival. Here, we investigated the prognostic values of peripheral CD8+CD28+ T cells and CD8+CD28− T cells in advanced NSCLC patients treated with chemo(radio)therapy and the impact of histological type on them.

**Methods:**

Of 232 registered advanced NSCLC patients, 101 treatment-naïve individuals were eligible and included in our study. Flow cytometry was used to evaluate CD8+CD28+ T cells, CD8+CD28− T cells, CD4+ CD25^hi^ T cells, B cells, natural killer cells, γδT cells, and natural killer T cells in patients’ peripheral blood.

**Results:**

The median follow-up time was 13.6 months. Fifty-nine (58.4%) patients died by the end of our study. Fifty-three of the 101 advanced NSCLC cases selected for our study were adenocarcinomas (ADs), and 48 were squamous cell carcinomas (SCCs). Multivariate analyses showed that increased levels of CD8+CD28+ T cells independently predicted favorable overall survival (OS) [hazard ratio (HR): 0.51, 95% confidence interval (CI) 0.30–0.89, P = 0.021] and progression-free survival (PFS) (HR: 0.66, 95% CI 0.37–0.93, P = 0.038) in ADs, but the prediction in SCCs was not statistically significant. In contrast, high levels of CD8+CD28− T cells independently predicted unfavorable OS (HR: 1.41, 95% CI 1.17–3.06, P = 0.035) and PFS (HR: 2.01, 95% CI 1.06–3.85, P = 0.029) in SCCs, but the prediction in ADs was not statistically significant. ADs had higher levels of CD4+CD25^hi^ T cells and CD8+CD28− T cells and lower NK cells (all P < 0.05) than SCCs.

**Conclusions:**

Our findings uncovered the prognostic values of peripheral CD8+CD28+ T cells and CD8+CD28− T cells in advanced NSCLC patients treated with chemo(radio)therapy, which could help to identify patients with poor outcomes and refine treatment strategies.

## Background

Among all lung cancer patients, more than 80% of patients have non-small cell lung cancer (NSCLC), which mainly consists of adenocarcinomas (ADs) and squamous cell carcinomas (SCCs) [1, 2]. Chemo(radio)therapy is the standard treatment for advanced NSCLC patients [3–5]. These advanced patients have diverse clinical outcomes [5]. Consequently, prognostic markers are needed to identify patients with poor outcomes and refine the treatment strategies for them. To date, some prognostic markers were investigated in advanced NSCLC, including positron emission tomography parameters, driver gene mutation, number of metastatic sites, interleukin-6, cell-free DNA, circulating tumor cells, inflammation parameters, and tumor-infiltrating lymphocytes (TILs) [6–16]. More noninvasive prognostic biomarkers are needed for advanced NSCLC patients with different histological types to identify cases with poor survival.

Several studies have revealed the differences in the expression of genes, methylation, and tumor immune microenvironment between lung AD and SCC [6, 17–21]. Faruki et al. [19] reported major differences in the tumor immune landscapes of the expression subtypes of lung AD and SCC. The immune cell expression of the proximal proliferative subtype (with serine/threonine kinase 11 (STK11) gene deletion, poor prognosis, and high proliferation) was low among ADs, whereas, the immune cell expression of the secretory subtype (with the genomic data of greater inflammatory response) was high among SCCs. Per Kinoshita et al. [6], different prognostic roles are played by TILs in AD and non-AD. Specifically, they identified a high ratio of forkhead box P3+ (FOXP3+) to CD4+ T cells and a low buildup of CD20+ B cells as worse factors of prognosis in AD patients. Fewer CD8+ T cells correlated with a negative outcome in non-AD. Thus, the histological type could impact the immune cells’ prognostic role in NSCLC.

Despite this advanced knowledge, the effect of histological type on the prognostic role of peripheral immune cells, collected by a rapid and noninvasive method, remains to be clarified. The anti-tumor effect of CD8+ T cells requires the activation of two co-stimulatory signals. Firstly, T cell receptors on CD8+ T cells recognize and combine with tumor antigens presented by antigen-presenting cells (APCs). Secondly, CD28, as another significant signal on CD8+ T cells, combines with B7 molecules on APCs. T cells are activated and exert immune responses to tumors when both signals are engaged [22, 23]. However, CD28 expression in CD8+ T cells may be down-regulated in patients with tumors because of tumor antigens’ chronic stimulation [24]. Finally, CD8+ T cells are less responsive to tumor antigens and lose control of tumor cells in cancer patients [24, 25].

Our previous study suggested that tumor progression in NSCLC patients undergoing radiotherapy could be predicted independently by peripheral regulatory T cells [26]. In this present study, we aimed to further clarify peripheral immune prognostic factors, especially for CD8+CD28+ T cells and CD8+CD28− T cells, in advanced lung ADs and SCCs.

## Methods

### Patients

Clinical stage III and IV patients with histologically confirmed NSCLC were selected for this study. Patients with targetable oncogenes [including anaplastic lymphoma kinase (ALK), epidermal growth factor receptor (EGFR), cMET, and Ki-ras (KRAS)] were excluded, as well as were patients with incomplete clinicopathological data, performance status (PS) > 2, hematological, renal, and liver diseases, general infection, and other tumors, and those who received immune-related drugs, including granulocyte-colony stimulating factor, steroids, and antilymphocyte globulin, during the 3 months preceding enrollment. Of 232 advanced NSCLC patients enrolled between April 2014 and April 2017, 101 treatment-naïve patients were eligible and included in our study. Fifty-eight age- and sex-matched healthy volunteers were chosen as control.

### Flow cytometry

Four milliliters of fresh blood were collected from healthy volunteers and patients during the 3 days preceding any anti-tumor treatments (chemo/radiation/immunotherapy/surgery) and stored in EDTA in anti-coagulant tubes. Peripheral leucocytes were stained with surface markers using the following specific anti-human monoclonal antibodies for 15 min in the dark, at room temperature: CD8 FITC, CD28 PE, CD45 PerCP, CD3 APC, CD4 FITC, CD25 APC, CD3 FITC, CD16+CD56 PE, CD19 APC, and γδ T Cell Receptor (TCR) PE to identify seven lymphocytes subsets: B cells (CD3−CD19+), natural killer (NK) cells [CD3−(CD16+56+)], γδT cells (CD3+γδTCR+), NKT cells [CD3+(CD16+56+)], CD4+CD25^hi^ T cells (CD4+CD25^hi^), CD8+CD28− T cells (CD3+CD8+CD28−), and CD8+CD28+ T cells (CD3+CD8+CD28+).

Next, red blood cell lysis was performed with Red Blood Cell lysing buffer (BD Biosciences; USA) for 10 min in the dark, at room temperature, followed by flow cytometry (BD Biosciences; USA) for the analysis of residual white blood cells. We used the FlowJo Version 10 data analysis software (FlowJo, Ashland, OR, USA) to determine the frequency of total lymphocytes for each lymphocyte subset. Representative figures showing the gating of each population are presented in Additional file 1: Figure S1.

### Data collection, treatment, and follow-up

We collected information on the stage of the disease, histology, tumor differentiation, smoking status, gender, age, and performance status as per the American Joint Committee on Cancer (AJCC-7 criteria [27]). Every patient was treated with cisplatin-based chemotherapy for 4–6 cycles. Among 75 stage IV patients, 11 received consolidated radiotherapy (60–66 Gy/30–33 fractions or 50 Gy/5 fractions) for lung lesions after chemotherapy. Stage III patients were subjected to concurrent chemoradiotherapy (60–66 Gy/30–33 fractions). Follow-up was performed regularly every 3 months and ended in October 2018.

### Statistical analysis

Cut-off values (high vs. low) of every immune cell were determined using the median values of the cells. In subgroup analyses, the median values of each group were used to determine cut-off values. Continuous parameters were presented as mean ± standard deviation. We used the Student’s t-test to compare immune cells between two groups. Univariate and multivariate Cox proportional hazards regression models were used for the evaluation of hazard ratios (HRs). Univariate-analyzed variables with *P *< 0.010 were examined further using multivariate analytics. The area under the receiver operating characteristic curve was used to evaluate immune cells’ predictive ability for patients’ survival. We defined PFS as the period from patient enrollment to disease relapse, tumor metastasis, death, or end or loss of follow-up. OS represented the time between patient enrollment and death, or end or loss of follow-up. We utilized the Kaplan–Meier curve for the estimation of patient survival. The log-rank test was use to compare survival between groups. The SPSS 23.0 software was used for Data analysis (SPSS Inc., Chicago, IL). We considered a *P*-value < 0.05 statistically significant.

## Results

### Demographics

The characteristics of the 101 advanced NSCLC patients enrolled in this study are shown in Additional file 2: Table S1. Among them, 42 patients were < 60 years old, and 59 were ≥ 60 years old; 65 were males, and 36 were females; 53 were ADs, and 48 were SCCs; 43 were non-smokers, and 58 were smokers; 26 were stage III, and 75 were stage IV; 30 were PS 0, 66 were PS 1, and 5 were PS 2. The median follow-up period amounted to 13.6 months, at the end of which 59 (58.4%) patients had died, and one patient had lost follow-up.

### Lymphocytes subsets and their prognostic value in 101 NSCLC cases

**Fig. 1:**
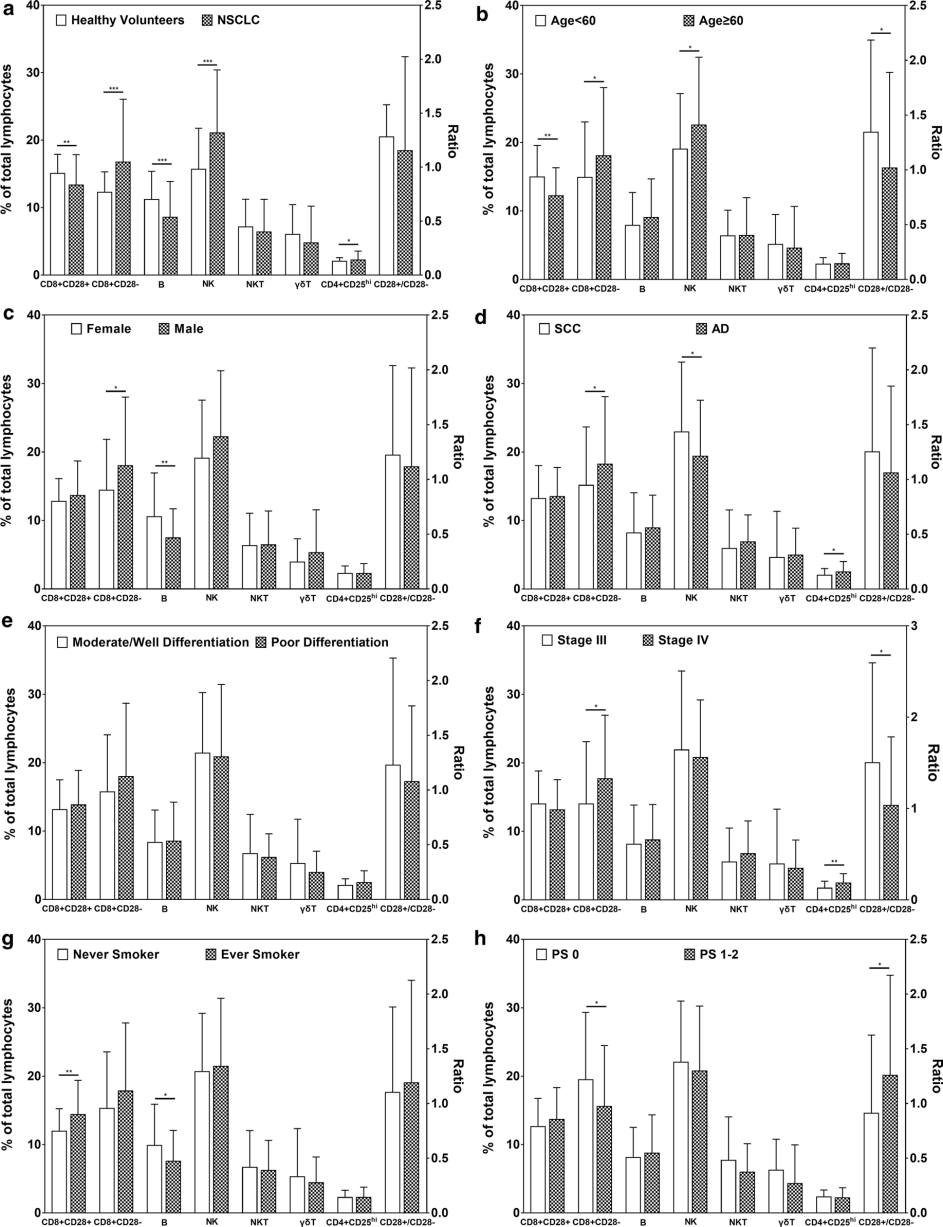
Comparing subsets of peripheral lymphocytes between patients with **a** advanced NSCLC and healthy volunteers, **b** age < 60 and age ≥ 60, **c** male and female, **d** ADs and SCCs, **e** well/moderate differentiation and poor differentiation, **f** stage III and stage IV, **g** ever smokers and never smokers, and **h** PS 0 and PS 1–2. Data are presented as mean ± SD. The Student’s t-test was used for comparison of immune cells between two groups. *P < 0.05, **P < 0.01, ***P < 0.001

The median count of the total lymphocytes in 101 NSCLC patients was 1.39 (0.52–3.83) × 10^9^/L. Figure 1 shows how lymphocytes subsets compare between patients with NSCLC and healthy volunteers, male and female, ADs and SCCs, stage III and stage IV, smokers and non-smokers, age < 60 and age ≥ 60, and PS 0 and PS 1–2. According to the results, NSCLC patients had lower CD8+CD28+ T cells and B cells (both P < 0.01) and slightly higher CD4+CD25^hi^ T cells, higher CD8+CD28− T cells, and NK cells (all P < 0.05) than healthy volunteers; ADs had slightly higher CD4+CD25^hi^ T cells, higher CD8+CD28− T cells and lower NK cells (all P < 0.05) than SCCs; stage IV patients had higher CD4+CD25^hi^ T cells and CD8+CD28− T cells than stage III patients (both P < 0.05); female patients had lower CD8+CD28− T cells and higher B cells than male patients (both P < 0.05); and smokers had lower B cells and higher CD8+CD28+ T cells than non-smokers (both P < 0.05).

**Fig. 2:**
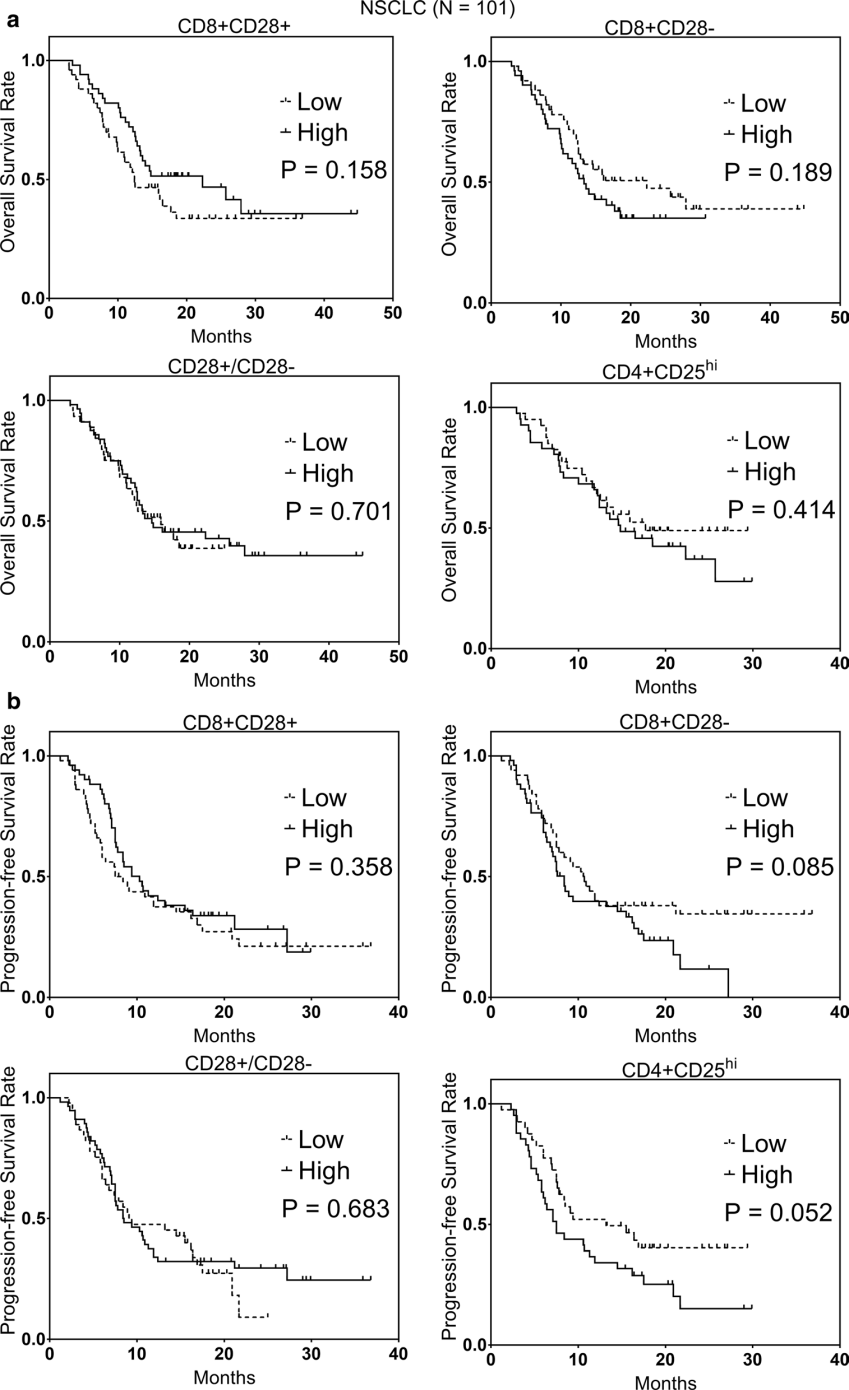
The Kaplan–Meier representation of **a** OS and **b** PFS between high and low levels of CD8+CD28− T cells, CD8+CD28+ T cells, CD28+/CD28− ratio, and CD4+CD25^hi^ T cells in 101 patients with advanced NSCLC

Additional file 1: Figure S2 shows the distribution and median values of lymphocytes subsets in 101 NSCLC patients. The median values of CD8+CD28+ T cells, CD8+CD28− T cells, B cells, NK cells, NKT cells, γδT cells, CD4+CD25^hi^ T cells, and CD28 +/CD28− ratios were 12.3 (6.1–28.4), 14.2 (3.3–42.9), 6.8 (1.6–25.6), 21.2 (3.2–53.6), 5.2 (1.0–26.3), 3.4 (0.3–42.8), 2.0 (0.4–10.0), and 0.9 (0.2–5.1), respectively. The Kaplan–Meier analysis was used to compare the OS and PFS of patients with high and low levels of immune cells and revealed no statistical difference of significance in the OS and PFS between groups (all P > 0.05), as shown in Fig. 2 and Additional file 1: Figure S3.

### The favorable prognostic role of high levels of CD8+CD28+ T cells in ADs

**Fig. 3:**
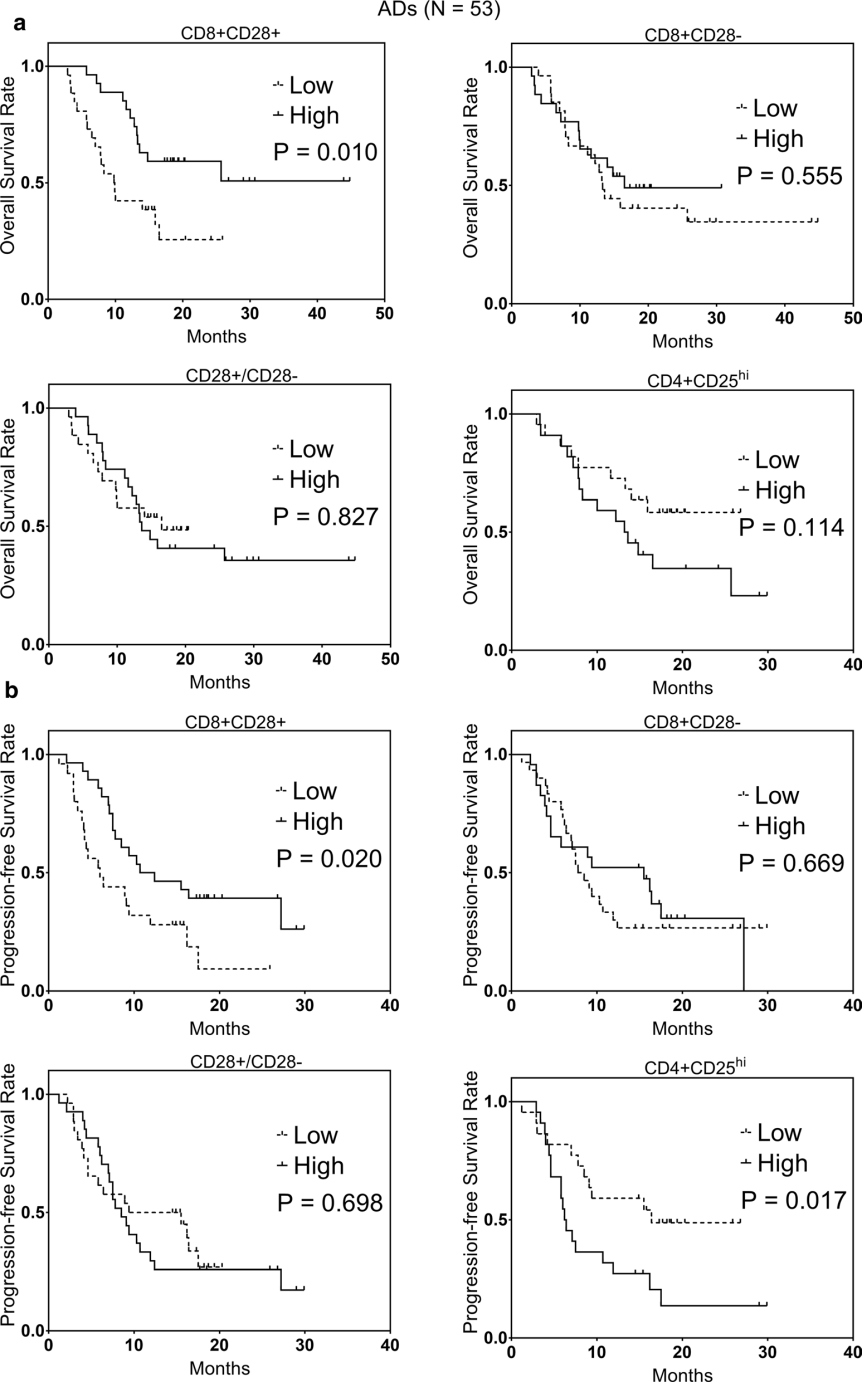
The Kaplan–Meier representation of **a** OS and **b** PFS between high and low levels of CD8+CD28− T cells, CD8+CD28+ T cells, CD28+/CD28− ratio, and CD4+CD25^hi^ T cells in 53 ADs

The Kaplan–Meier analysis showed that ADs with high CD8+CD28+ T cells had better OS and PFS than ADs with low CD8+CD28+ T cells (P = 0.010 and 0.020, respectively, Fig. 3). In addition, ADs with high CD4+CD25^hi^ T cells had worse PFS than ADs with low CD4+CD25^hi^ T cells (P = 0.017, Fig. 3). The univariate analyses for the survival of ADs revealed that high levels of CD8+CD28+ T cells predicted favorable OS (HR: 0.52, 95% CI 0.25–0.92, P = 0.013, Table 1) and PFS (HR: 0.59, 95% CI 0.29–0.96, P = 0.016, Table 1). Furthermore, increased levels of CD4+CD25^hi^ T cells predicted an unfavorable PFS (HR: 1.96, 95% CI 1.22–2.81, P = 0.011, Table 1). Other immune cells did not correlate significantly with survival in our study (Additional file 1: Figure S4, Table 1).

**Table 1:** Univariate and multivariate analyses for survival of adenocarcinoma patients

Variables	Overall survival		Progression-free survival	
	HR (95% CI)	P	HR (95% CI)	P
A (univariate)				
Gender				
Male	1.88 (0.89–3.97)	0.094	2.16 (1.10–4.23)	0.025
Female	1		1	
Age (years)				
≥ 60	1.23 (0.60–2.54)	0.565	1.12 (0.58–2.13)	0.731
< 60	1		1	
Smoking status				
Ever smokers	1.73 (0.84–3.55)	0.136	1.60 (0.84–3.04)	0.147
Never smokers	1		1	
cStage				
IV	1.05 (0.25–4.45)	0.940	0.76 (0.23–2.49)	0.658
III	1		1	
Tumor differentiation				
Poor	1.90 (0.88–4.11)	0.101	1.75 (0.89–3.45)	0.103
Well/moderate	1		1	
Performance status				
1–2	1.68 (0.64–4.41)	0.291	1.97 (0.82–4.73)	0.129
0	1		1	
B	0.99 (0.92–1.07)	0.818	0.93 (0.86–1.06)	0.677
NK	1.01 (0.96–1.05)	0.892	0.96 (0.91–1.02)	0.645
NKT	0.87 (0.58–1.09)	0.583	0.68 (0.36–1.07)	0.328
γδT	0.94 (0.85–1.05)	0.298	0.95 (0.86–1.04)	0.295
CD8+CD28+	0.52 (0.25–0.92)	0.013	0.59 (0.29–0.96)	0.016
CD8+CD28−	0.93 (0.79–1.12)	0.349	0.98 (0.95–1.02)	0.314
CD28+/CD28−	1.08 (0.69–1.68)	0.735	1.00 (0.68–1.47)	0.991
CD4+CD25^hi^	1.57 (0.88–2.46)	0.153	1.96 (1.22–2.81)	0.011
B (multivariate)				
Gender	1.92 (0.82–4.49)	0.133	2.12 (0.99–4.57)	0.055
CD8+CD28+	0.51 (0.30–0.89)	0.021	0.66 (0.37–0.93)	0.038
CD4+CD25^hi^	–	–	1.53 (1.19–1.97)	0.031

P-values for multivariate analyses are adjusted. The HR and 95% CI were reported for 1 SD increase for immune cells

According to multivariate analysis findings, high levels of CD8+CD28+ T cells independently predicted favorable OS (HR: 0.51, 95% CI 0.30–0.89, P = 0.021, Table 1) and PFS (HR: 0.66, 95% CI 0.37–0.93, P = 0.038, Table 1) in ADs. The areas under receiver operating characteristic curves (AUCs) were estimated for risk models with only CD8+CD28+ T cells for OS and PFS in ADs were 0.687 and 0.655, respectively. Also, high CD4+CD25^hi^ T cells independently predicted an unfavorable PFS (HR: 1.53, 95% CI 1.19–1.97, P = 0.031, Table 1).

### The unfavorable prognostic role of increased levels of CD8+CD28− T cells in SCCs

**Fig. 4:**
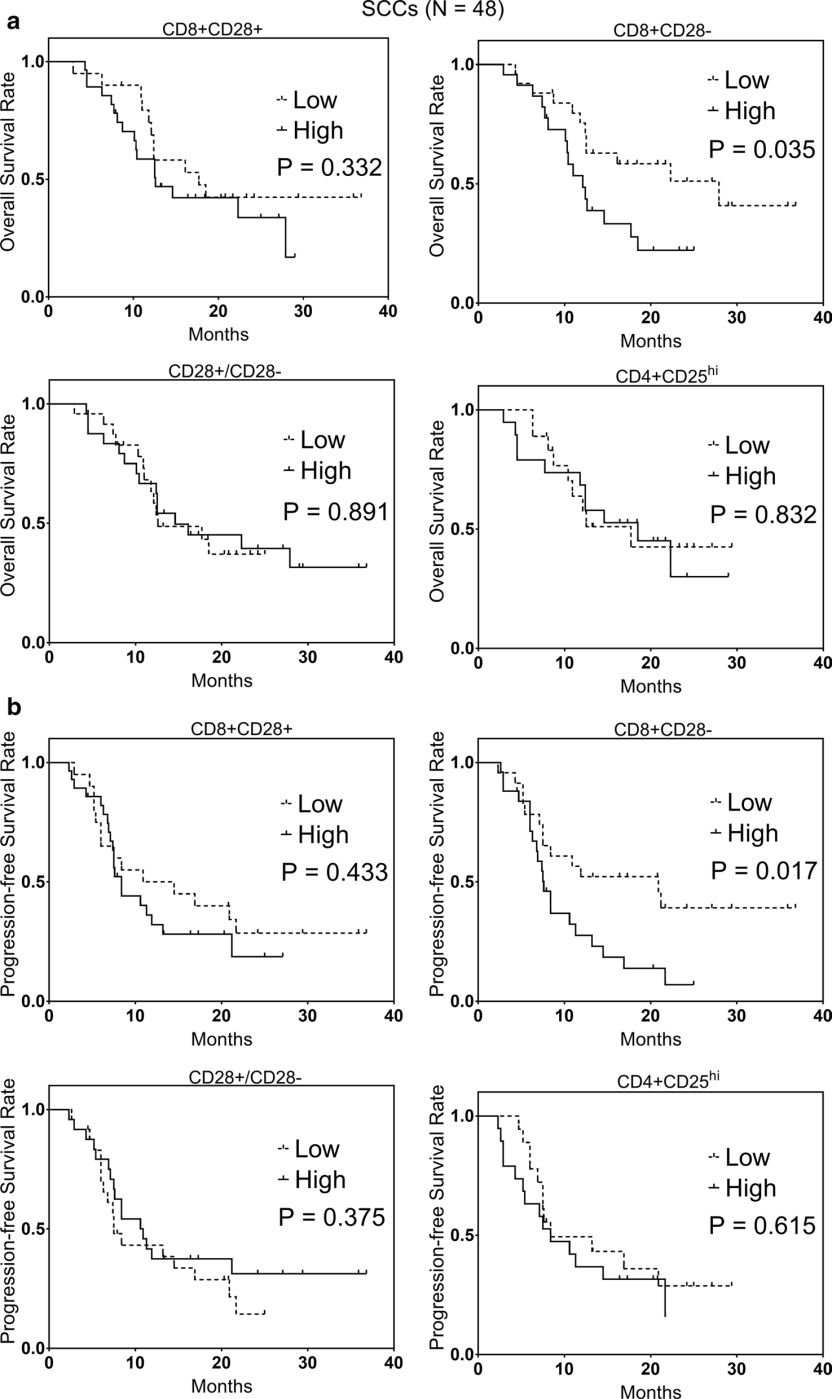
The Kaplan–Meier representation of **a** OS and **b** PFS between high and low levels of CD8+CD28− T cells, CD8+CD28+ T cells, CD28+/CD28− ratio, and CD4+CD25^hi^ T cells in 48 SCCs

The Kaplan–Meier analysis showed that SCCs with high CD8+CD28− T cells had worse OS and PFS than SCCs with low CD8+CD28− T cells (P = 0.035 and 0.017, respectively, Fig. 4). Results from univariate analyses showed that high CD8+CD28− T cells predicted unfavorable OS (HR: 2.01, 95% CI 1.16–4.05, P = 0.039, Table 2) and PFS (HR: 2.12, 95% CI 1.07–4.36, P = 0.023, Table 2). No other immune cells were found to be linked significantly to survival in our study (Additional file 1: Figure S5, Table 2).

**Table 2:** Univariate and multivariate analyses for survival of squamous cell carcinoma patients

Variables	Overall survival		Progression-free survival	
	HR (95% CI)	P	HR (95% CI)	P
A (univariate)				
Gender				
Male	1.50 (0.56–3.96)	0.414	0.97 (0.42–2.25)	0.954
Female	1		1	
Age (years)				
≥ 60	0.47 (0.21–1.05)	0.069	0.49 (0.23–1.04)	0.065
< 60	1		1	
Smoking status				
Ever smokers	1.08 (0.46–2.56)	0.852	0.81 (0.37–1.73)	0.581
Never smokers	1		1	
cStage				
IV	1.22 (0.58–2.56)	0.597	1.15 (0.58–2.27)	0.681
III	1		1	
Tumor differentiation				
Poor	1.30 (0.58–2.91)	0.52	1.29 (0.60–2.75)	0.512
Well/moderate	1		1	
Performance status				
1–2	1.72 (0.72–4.06)	0.215	1.30 (0.63–2.68)	0.471
0	1		1	
B	1.29 (0.91–2.03)	0.328	1.17 (0.96–1.82)	0.532
NK	0.97 (0.57–1.82)	0.165	0.92 (0.62–1.56)	0.181
NKT	1.19 (0.73–1.65)	0.720	1.21 (0.86–1.76)	0.824
γδT	0.65 (0.35–1.36)	0.611	0.78 (0.43–1.44)	0.955
CD8+CD28+	1.01 (0.94–1.10)	0.749	0.99 (0.93–1.07)	0.902
CD8+CD28−	2.01 (1.16–4.05)	0.039	2.12 (1.07–4.36)	0.023
CD28+/CD28−	0.96 (0.68–1.37)	0.863	0.87 (0.61–1.22)	0.414
CD4+CD25^hi^	1.17 (0.68–2.04)	0.570	1.28 (0.78–2.10)	0.331
B (multivariate)				
Age (years)	0.47 (0.21–1.05)	0.064	0.48 (0.22–1.02)	0.057
CD8+CD28−	1.41 (1.17–3.06)	0.035	2.01 (1.06–3.85)	0.029

P-values for multivariate analyses are adjusted. The HR and 95% CI were reported for 1 SD increase for immune cells

Per multivariate analyses findings, CD8+CD28− T cells independently predicted unfavorable OS (HR: 1.41, 95% CI 1.17–3.06, P = 0.035, Table 2) and PFS (HR: 2.01, 95% CI 1.06–3.85, P = 0.029, Table 2) in SCCs. AUCs were estimated for risk models with only CD8+CD28− T cells for OS and PFS in SCCs were 0.689 and 0.713, respectively.

## Discussion

A previous investigation showed already that the prognostic value of TILs fluctuates depending on the histological type [6]. Here, we present additional information on this issue in terms of peripheral lymphocytes subsets. High levels of peripheral CD8+CD28+ T cells presented favorable prognosis in ADs. Moreover, increased levels of CD8+CD28− T cells independently predicted poor survival in SCCs. ADs also exhibited higher CD4+CD25^hi^ T cells and CD8+CD28− T cells and lower NK cells than SCCs.

In NSCLC, higher peripheral CD8+CD28− T cells, CD4+CD25+FOXP3+ regulatory T cells, CTLA-4+ cells (in both CD4+and CD8+T cells), proliferating CD8(+) T cells, and lower CD4+T cells and CD4/CD8 ratios have been observed when compared with healthy controls [26, 28–30]. Fewer CD8+ and CD4+ tumor infiltrating lymphocytes (TILs) were found within tumor cell clusters when compared with the stromal compartment in NSCLC [31]. TILs and tumor-infiltrating regulatory T cells were detected in 83% and 51%, respectively, of early-stage NSCLC patients [32].

Song et al. [33] revealed that high levels of peripheral CD8+CD28+T cells are linked to prolonged PFS, whereas high CD8+CD28− T cells correlate with shortening PFS in metastatic breast cancer patients. Additionally, the same investigation reported increased CD8+CD28− T cells and CD4+CD25^hi^ T cells and decreased CD8+CD28+ T cells in metastatic breast cancer patients when compared with healthy volunteers. Another study reported increased CD8+CD28− T cells and CD4+CD25^hi^ T cells in advanced lung cancer patients [29]. Consistent with these investigations, we now report similar results in advanced NSCLC.

Although our results are similar to those of previous findings, those studies did not examine the prognostic values of immune cells in NSCLC. Most evaluations have centered primarily on the role of CD8+T cells in peripheral blood and the tumor microenvironment in various tumors, with no further classification of CD28 cells into CD28+ and CD28− subgroups [30, 31, 34–40]. CD28 is a co-stimulatory molecule that is important for the activation of CD8+T cells, which play an important role in anti-tumor immunity [41–44]. Recent studies have also proven that PD-1-targeted therapies’ salvaging of exhausted CD8 T cells depends on CD28 [43]. On the contrary, the loss of expression of CD28 leads to CD8 T cells losing their cytotoxic function and inhibiting the proliferation of T cells [44]. Hence, we speculated that CD28 expression in CD8+T cells influences their anti-tumor immune response in NSCLC patients, and, consequently, impact patients’ survival.

To the best of our knowledge, our study is the first to report the prognostic value of peripheral CD8+CD28+ T cells and CD8+CD28− T cells in advanced NSCLC based on histological types. We showed the existing correlation between increased peripheral CD8+CD28+ T cells and prolonged survival in ADs, which is consistent with the anti-tumor function of CD8+CD28+ T cells [22, 45]. We demonstrated further that high CD8+CD28− T cells predicted an unfavorable survival in SCCs, which may because of the loss of CD28 on CD8+ T cells and consequent inhibitory effect of them [46]. However, we found that the prognostic value of CD8+CD28+ T cells in SCCs and CD8+CD28− T cells in ADs did not reach statistical significance, which may have arisen from the differences in tumor immunity, gene expression, and methylation between ADs and SCCs [6, 17–20].

Although our study produced promising results, it still has its limitations. Firstly, although diseases that could impact peripheral immune cells were excluded, some undetected or unreported ailments could have slipped through our selection criterion. Secondly, patients’ systematic immune functioning could have been influenced by their daily eating, activity, and sleeping. Thirdly, the sample size of our investigation was relatively small. Finally, different p-values may have resulted from the different high/low stratification of immune cells and different patients between ADs and SCCs. Thus, a larger and more uniform patient cohort should potentially validate these results.

## Conclusions

We uncovered the prognostic values of peripheral CD8+CD28+ and CD8+CD28− T cells in advanced NSCLC patients treated with chemo(radio)therapy. High levels of peripheral CD8+CD28+ T cells showed favorable prognosis in ADs. Furthermore, increased levels of CD8+CD28− T cells independently predicted poor survival in SCCs. ADs also had higher CD4+CD25^hi^ T cells and CD8+CD28− T cells and lower NK cells than SCCs. These results may help to identify advanced NSCLC patients with poor outcomes and refine treatment strategies.

## Supplementary information

**Additional file 1: Figure S1.** Representative flow cytometry plots and gating. **Figure S2.** The distribution and median values of lymphocytes subsets in (A) 101 NSCLC patients, (B) 53 ADs, and (C) 48 SCCs. **Figure S3.** Comparing (A) OS and (B) PFS between high and low levels of B cells, NK cells, γδT cells, and NKT cells in 101 patients with advanced NSCLC. **Figure S4.** Comparing (A) OS and (B) PFS between high and low levels of B cells, NK cells, γδT cells, and NKT cells in 53 ADs. **Figure S5.** Comparing (A) OS and (B) PFS between high and low levels of B cells, NK cells, γδT cells, and NKT cells in 48 SCCs.

**Additional file 2: Table S1.** Demographics.

## Data Availability

All data included in our study are shown in our manuscript.

## Abbreviations

PD-1: programmed death-1
AD: adenocarcinomas
OS: overall survival
SCC: squamous cell carcinoma
PFS: progression-free survival
NSCLC: non-small cell lung cancer
