# Supplementary material for: Prognostic significance of peripheral CD8+CD28+ and CD8+CD28− T cells in advanced non-small cell lung cancer patients treated with chemo(radio)therapy
Source: J Transl Med. 2019 Oct 17;17:344. doi: 10.1186/s12967-019-2097-7 (PMC6796409; doi:10.1186/s12967-019-2097-7)
Supplement: Supplementary file 1 — Additional file 1: Figure S1. Representative flow cytometry plots and gating. Figure S2. The distribution and median values of lymphocytes subsets in (A) 101 NSCLC patients, (B) 53 ADs, and (C) 48 SCCs. Figure S3. Comparing (A) OS and (B) PFS between high and low levels of B cells, NK cells, γδT cells, and NKT cells in 101 patients with advanced NSCLC. Figure S4. Comparing (A) OS and (B) PFS between high and low levels of B cells, NK cells, γδT cells, and NKT cells in 53 ADs. Figure S5. Comparing (A) OS and (B) PFS between high and low levels of B cells, NK cells, γδT cells, and NKT cells in 48 SCCs. [file 12967_2019_2097_MOESM1_ESM.docx]

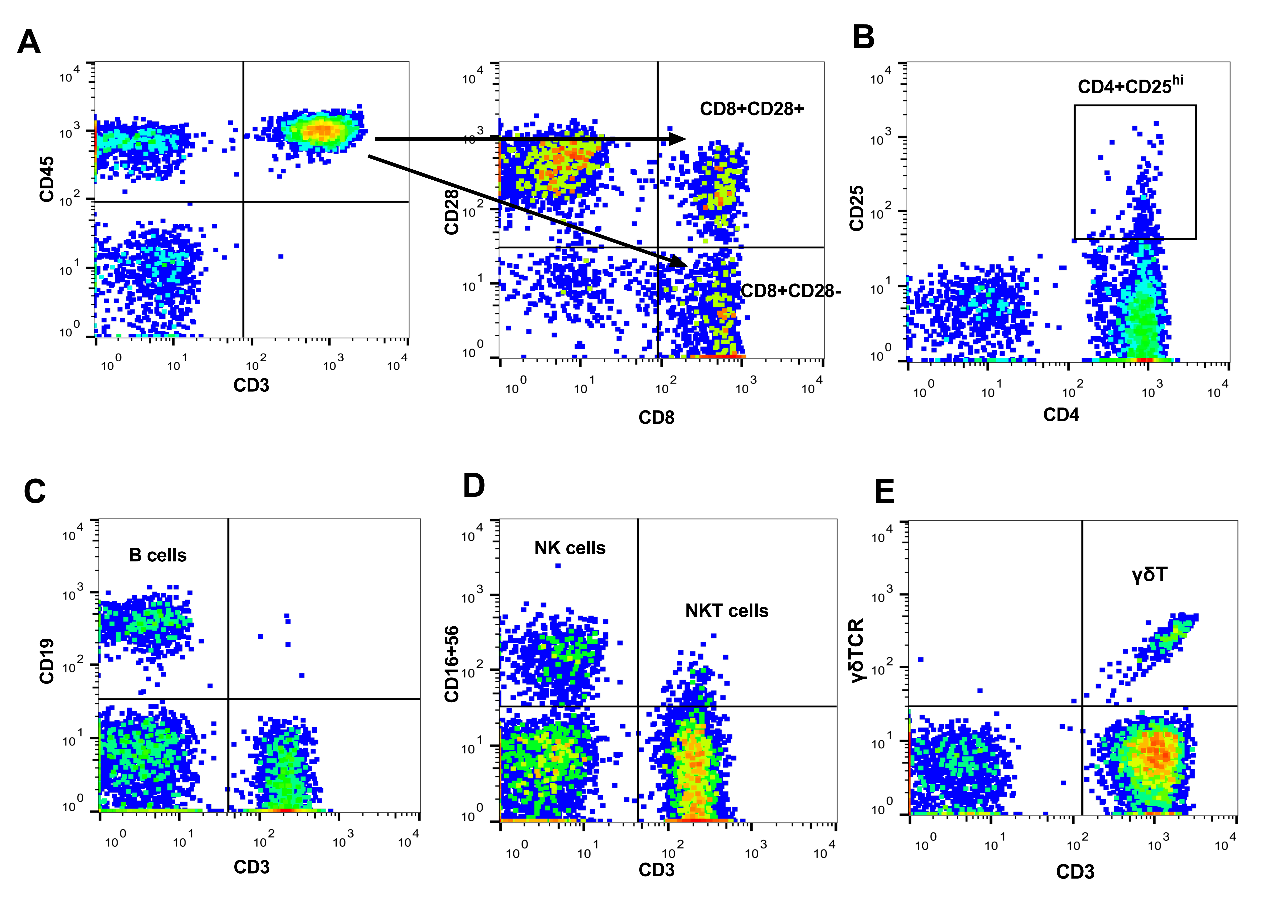
 **Additional file 1: Figure S1.** Representative flow cytometry plots and gating.


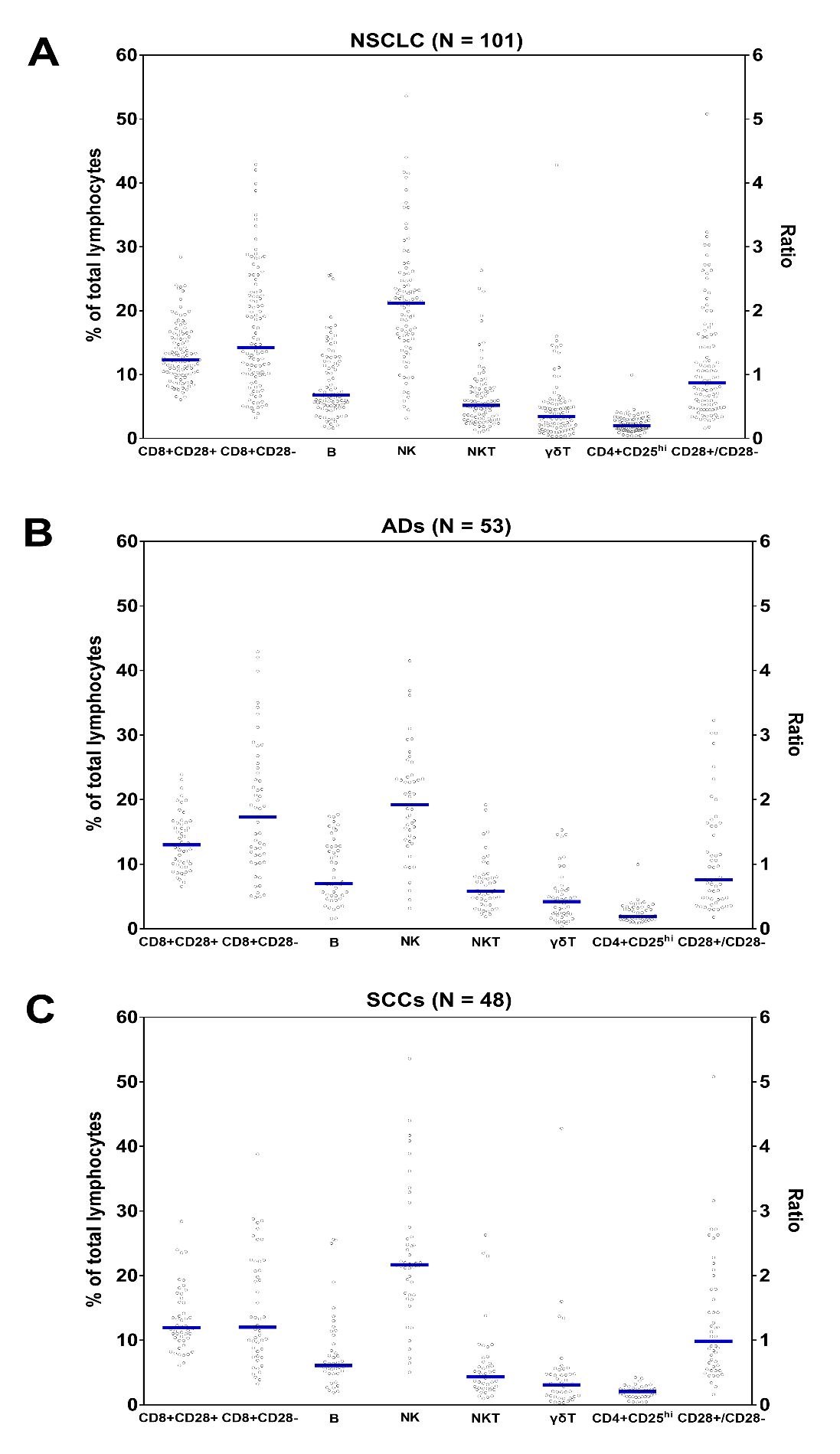


**Additional file 1: Figure S2.** The distribution and median values of lymphocytes subsets in (A) 101 NSCLC patients, (B) 53 ADs, and (C) 48 SCCs.


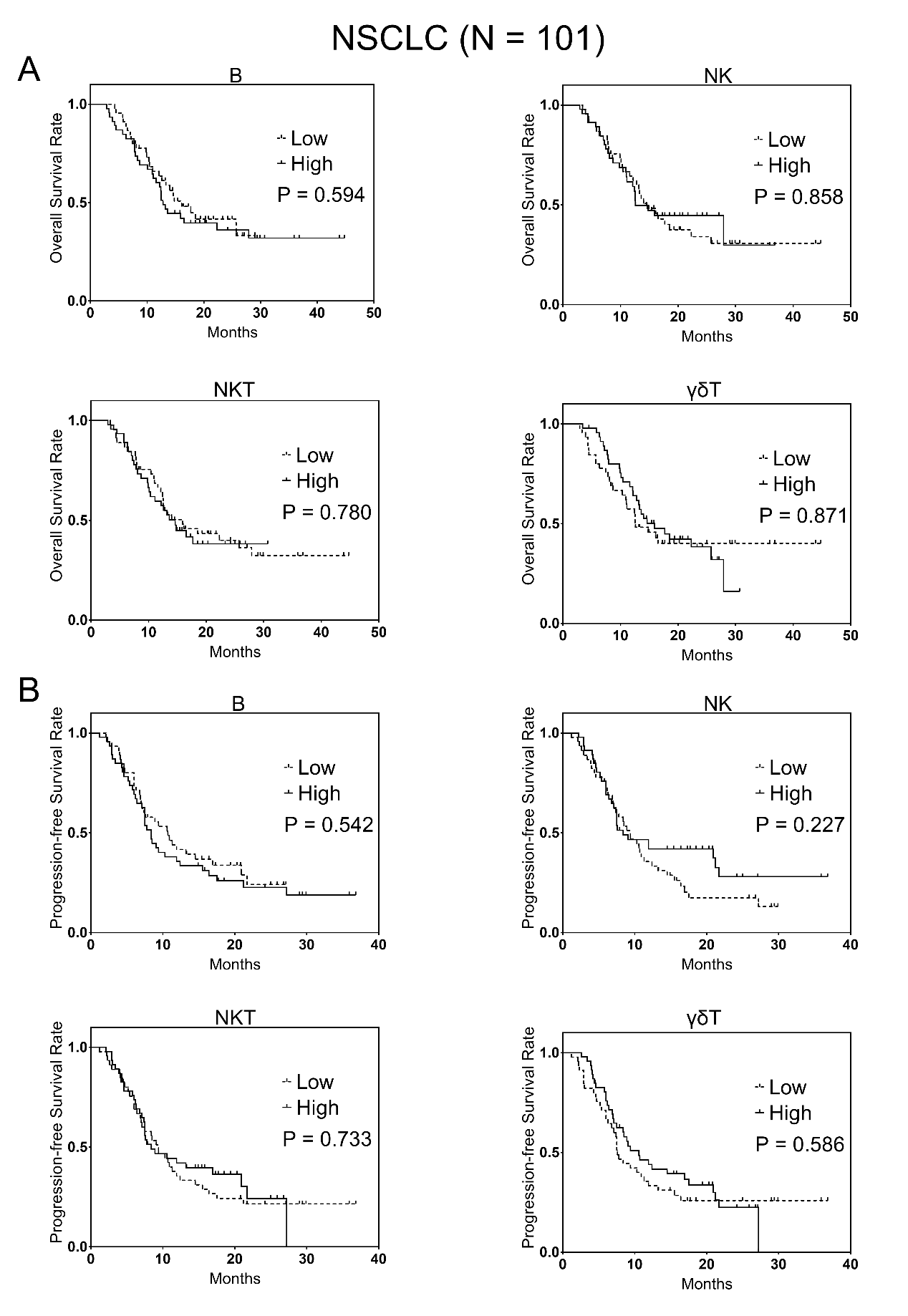


**Additional file 1: Figure S3.** Comparing (A) OS and (B) PFS between high and low levels of B cells, NK, γδT, and NKT in 101 patients with advanced NSCLC.


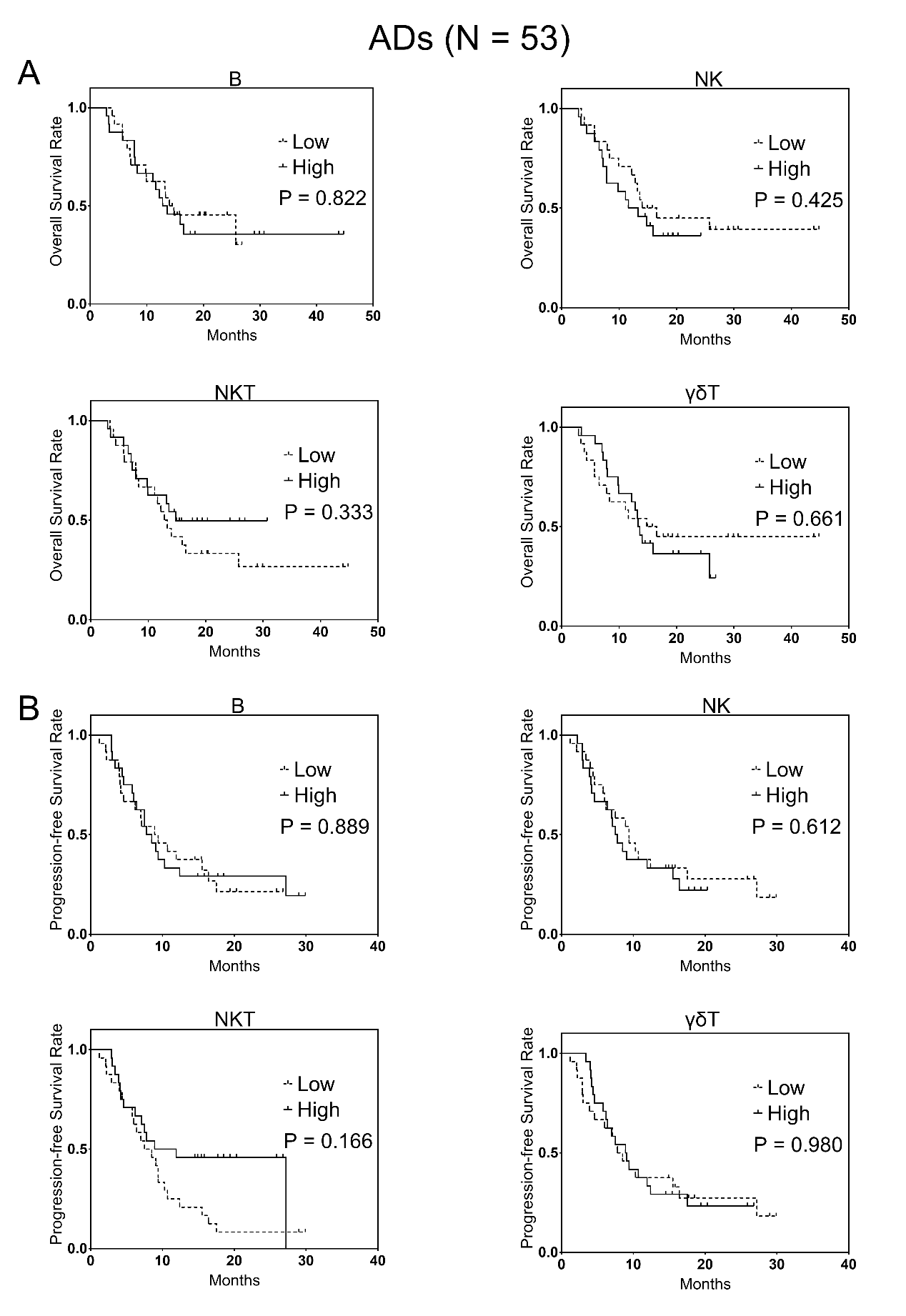


**Additional file 1: Figure S4.** Comparing (A) OS and (B) PFS between high and low levels of B cells, NK, γδT, and NKT in 53 ADs.


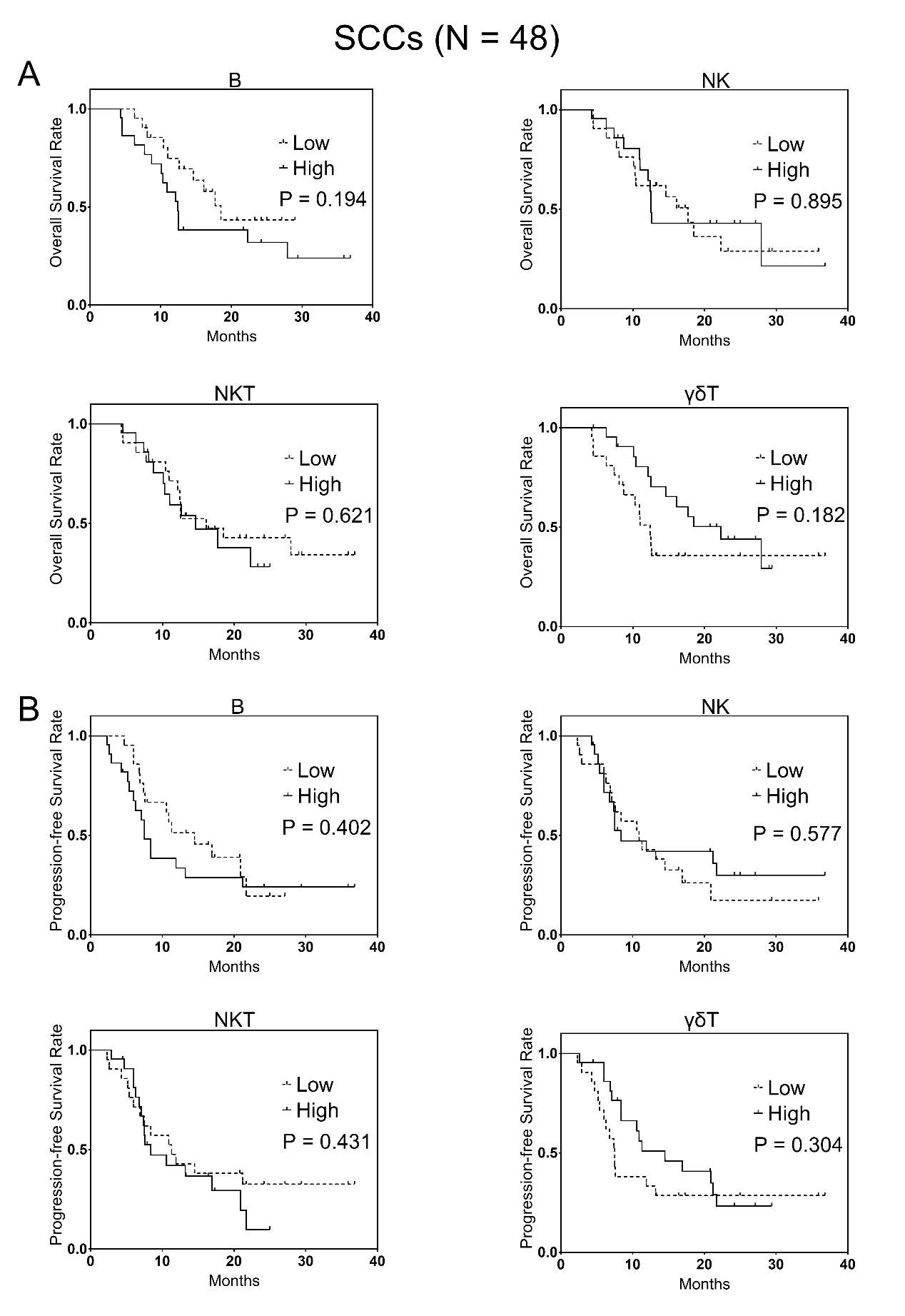


**Additional file 1: Figure S5.** Comparing (A) OS and (B) PFS between high and low levels of B cells, NK, γδT, and NKT in 48 SCCs.
