# Supplementary material for: Prognostic significance of peripheral CD8+CD28+ and CD8+CD28− T cells in advanced non-small cell lung cancer patients treated with chemo(radio)therapy
Source: J Transl Med. 2019 Oct 17;17:344. doi: 10.1186/s12967-019-2097-7 (PMC6796409; doi:10.1186/s12967-019-2097-7)
Supplement: Supplementary file 2 — Additional file 2: Table S1. Demographics. [file 12967_2019_2097_MOESM2_ESM.docx]

**Additional file 2: Table S1** Demographics

| Variables | N | (%) |
| --- | --- | --- |
| Gender |  |  |
| Male | 65 | 64.4 |
| Female | 36 | 35.6 |
| Age (years) |  |  |
| <60 | 42 | 41.6 |
| ≥60 | 59 | 58.4 |
| Histology |  |  |
| Adenocarcinoma | 53 | 52.5 |
| Squamous cell carcinoma | 48 | 47.5 |
| Smoking status |  |  |
| Never Smoker | 43 | 42.6 |
| Ever Smoker | 58 | 57.4 |
| cStage |  |  |
| III | 26 | 25.7 |
| IV | 75 | 74.3 |
| Tumor differentiation |  |  |
| Poor | 38 | 37.6 |
| Moderate | 53 | 52.5 |
| Well | 3 | 3.0 |
| Unknown | 7 | 6.9 |
| Performance Status |  |  |
| 0 | 30 | 29.7 |
| 1 | 66 | 65.3 |
| 2 | 5 | 5.0 |
